# EEG response varies with lesion location in patients with chronic stroke

**DOI:** 10.1186/s12984-016-0120-2

**Published:** 2016-03-02

**Authors:** Wanjoo Park, Gyu Hyun Kwon, Yun-Hee Kim, Jong-Hwan Lee, Laehyun Kim

**Affiliations:** Center for Bionics, Korea Institute of Science and Technology, Seoul, 02792 Korea; Department of Brain and Cognitive Engineering, Korea University, Seoul, 02841 Korea; Graduate School of Technology and Innovation Management, Hanyang University, Seoul, 04763 Korea; Department of Physical and Rehabilitation Medicine, Center for Prevention and Rehabilitation, Heart Vascular and Stroke Institute, Samsung Medical Center Sungkyunkwan University School of Medicine, Seoul, 06351 Korea; Samsung Advanced Institute for Health Science and Technology, Sungkyunkwan University, Seoul, 06351 Korea; Department of HCI & Robotics, University of Science and Technology, Daejeon, 34113 Korea

**Keywords:** Brain-computer interface, EEG, Rehabilitation, Stroke

## Abstract

**Background:**

Brain activation differs according to lesion location in functional magnetic resonance imaging (fMRI) studies, but lesion location-dependent electroencephalographic (EEG) alterations are unclear. Because of the increasing use of EEG-based brain-computer-interface rehabilitation, we examined lesion location-dependent EEG patterns in patients with stroke while they performed motor tasks.

**Methods:**

Twelve patients with chronic stroke were divided into three subgroups according to their lesion locations: supratentorial lesions that included M1 (SM1+), supratentorial lesions that excluded M1 (SM1-), and infratentorial (INF) lesions. Participants performed three motor tasks [active, passive, and motor imagery (MI)] with supination and grasping movements. The hemispheric asymmetric indexes, which were calculated with laterality coefficients (LCs), the temporal changes in the event-related desynchronization (ERD) patterns in the bilateral motor cortex, and the topographical distributions in the 28-channel EEG patterns around the supplementary motor area and bilateral motor cortex of the three participant subgroups were compared with those of the 12 age-matched healthy controls.

**Results:**

The SM1+ group exhibited negative LC values in the active and MI motor tasks, while the other patient subgroups exhibited positive LC values. Negative LC values indicate that the ERD/ERS intensity of the ipsilateral hemisphere is higher than the contralateral hemisphere, whereas positive LC values indicate that the ERD/ERS intensity of the contralateral hemisphere is higher than the ipsilateral hemisphere. The LC values of SM1+ and healthy controls differed significantly (rank-sum test, *p* < 0.05) in both the supination and grasping movements in the active task. The three patient subgroups differed distinctly from each other in the topography analysis.

**Conclusions:**

The hemispheric asymmetry and topographic characteristics of the beta band power patterns in the patients with stroke differed according to the location of the lesion, which suggested that EEG analyses of neurorehabilitation should be implemented according to lesion location.

**Electronic supplementary material:**

The online version of this article (doi:10.1186/s12984-016-0120-2) contains supplementary material, which is available to authorized users.

## Background

Stroke, which is the leading cause of adult neurological disabilities in most countries [1], typically damages particular regions of a patient’s brain and results in functional impairments [2]. These impairments vary depending on the location of the lesion. For instance, motor impairments are due to damage to the motor-related cortical regions [3, 4], cognitive deficits are usually associated with infarctions in the left anterior and posterior cerebral artery territories [5], and poststroke depression is correlated more with left frontal brain injuries than with lesions located in other areas [6, 7].

The process underlying the recovery of impaired motor functions after stroke involves brain plasticity, in which motor rehabilitation therapy stimulates new neural connections and enhances cortical reorganization in order to recover normal motor function [8, 9]. As a result, the undamaged areas of the nervous system take over the functions of the damaged areas [10].

Previous studies have shown that the recovery of motor function is influenced by lesion location. In a longitudinal study, Feydy et al. have shown that motor recovery is dependent on whether M1 is included in the lesion area [11]. Schiemanck et al. reported that the recovery of hand motor function in patients with internal capsule lesions had a significantly lower probability of recovery than that in patients with the cortical, subcortical, or corona radiata lesions [12]. Shelton et al. analyzed 41 post-stroke patients to investigate the effects of lesion location on upper limb motor recovery [4]. They found that the probability of recovery of isolated upper limb motor function decreases progressively with lesion location such as in the cortex, corona radiata, and posterior limbs of the internal capsule.

Neural stimulation studies have been beneficial to understand the reason that motor impairment and recovery are dependent on lesion location. As an example, transcranial magnetic stimulation (TMS) has been useful for exploring the neural mechanisms of motor function after stroke [13]. A TMS study reported that lesions in cortical or subcortical areas affected intracortical inhibitory properties [14].

In addition to motor recovery, brain activation is affected by lesion location. Using magnetic resonance imaging (MRI), Alexander et al. demonstrated that damage to the posterolateral putamen is associated with temporal gait asymmetry [15]. These findings suggest that damage to the inferior portion of the posterolateral putamen is associated with asymmetrical ambulation in the chronic stage of stroke recovery. Luft et al. recruited four groups (patients with cortical, subcortical, and brainstem stroke lesions and healthy volunteers), and functional MRI (fMRI) data were compared across these groups to investigate the brain activation of the participants during knee movement. They concluded that neural adaptation in brain networks was dependent on lesion location [16]. In an fMRI study of the upper limbs performed by Luft et al., the patients were divided into cortical and subcortical groups based on lesion location, and their brain activation was compared with that of healthy controls (HCs). The cortical stroke group showed less brain activation, whereas patients with subcortical lesions showed greater overall brain activation than the HCs [17].

In these fMRI studies, the brain activation patterns differed according to the lesion location. However, no studies have investigated the alterations in electroencephalography (EEG) responses according to lesion location. In light of technical advancement of EEG-based brain-computer interface (BCI) rehabilitation approaches [18, 19], a study to address this issue is urgently needed.

In our previous study, we investigated the levels of cognitive engagement of stroke patients by examining their brain activities while they performed active and passive hand movements [20]. We observed that active movement induced stronger event-related desynchronization (ERD) in the beta band compared to passive movement. These results showed that the beta band power patterns are associated with the level of motor engagement. However, in these studies, the lesion location of the patients had not been considered in the EEG data analysis.

In this study, we evaluated our hypothesis that the EEG patterns of patients with chronic stroke differed according to lesion location. The patients were divided into the three groups according to the location of their lesion: (1) patients with supratentorial lesions that included M1, (2) patients with supratentorial lesions that excluded M1, and (3) patients with infratentorial lesions. The three patients groups and HCs were compared to each other in terms of ERD power change in time, ERD topography in mu and beta bands, and the corresponding laterality coefficient (LC). The ERD and event-related synchronization (ERS) phenomenon are well known to be associated with motor movement and has been used to evaluate brain activities in BCI-based motor rehabilitation studies [20–22]. The LC of the ERD/ERS power of stroke patients is affected by brain damage. In general, healthy subjects show strong brain activation in the brain regions contralateral to the moving hand. However, when chronic stroke patients with damage to the brain regions controlling motor functions move their affected hand, they show brain activation in both hemispheres: weak activity in the ipsilesional (i.e. contralateral) regions, as expected, and strong activity in the contralesional (i.e. ipsilateral) regions. In stroke patients, neuroplasticity influenced the contralesional regions to take over some of the motor function of the lesioned area compromised by the brain injury [23–25]. Thus, the LC may be a good metric to evaluate the brain activation according to lesion location. Therefore, we expect that using both the ERD magnitude and LC metrics will lead to a better understanding of neural activities according to lesion location in stroke patients. In a previous study, Gong et al. have shown that patients with stroke exhibit different LC patterns of event-related potentials while performing motor imagery tasks compared with those of HCs [26]. Kaiser et al. also have investigated the relationship between the LC of ERS and motor function ability [23]. However, these studies did not systematically report the changes in the EEG LC patterns depending on the distinct lesion location.

## Methods

### Subjects

Twelve patients with chronic stroke (9 males, 3 females; mean ± SD age, 54.0 ± 6.6 years) participated in this study. All of the participants had a single stroke, exhibited unilateral motor problems in the upper extremities/limbs that continued for at least 3 months after their stroke, and were aged between 45 and 70 years old. Patients with cognitive disorders that rendered them unable to understand the task instructions and/or those with orthopedic disorders that led to amputation or joint contraction were excluded. The mean ± SD Fugl-Meyer Assessment scores were 47.3 ± 9.2 and 64.8 ± 9.2 for the affected and unaffected sides, respectively. Lower scores indicate more severe impairment. The patients did not have any history of neurological illness. The characteristics of these patients are provided in Table 1. Grasp strength, Purdue Pegboard Test, and Fugl-Meyer Assessment (FMA) have been used to evaluate motor functions of patients with stroke during rehabilitations phases [27–29]. More specifically, grasp strength shows the physical strength of the hand (clinical norms for the 55–59 years age group: men: right hand, 45.8; left hand, 37.7; women: right hand, 25.9, left hand 21.4 [kg]), Purdue Pegboard Test indicates the delicate control ability of the hand function (norms for the 55–59 years age group: men: right hand, 19.2; left hand, 21.0; women: right hand, 17.8, left hand 19.4), and FMA is generally used to evaluate the upper-limb functions for volitional movement ranges and reflex activities (scored on a scale of 0 and 66). Patients have significant differences in hand function between affected and unaffected hands in Grasping strength, Purdue Pegboard Test, and FMA (rank sum test, *p* < 0.01). These scores were used as exclusion criteria for patients with severe impairment (0 to 20 FMA score), and all participants in the moderate (21 to 50 score) or mild (51 to 66 score) categories, who were able to perform the motor tasks, were included [29]. A radiologist assessed and categorized lesion location based on the MRI data: (1) supratentorial lesions that included M1 (hereafter, SM1+), (2) supratentorial lesions that excluded M1 (SM1-), and (3) infratentorial (INF) lesions. SM1+ indicates a cortico-subcortical lesion and damaged M1, whereas SM1- indicates a subcortical lesion without M1 damage. The lesions of the SM1+ and SM1- groups are located in the supratentorial area while those of the INF group are in the infratentorial area. In addition to the patients with stroke, twelve age- and sex-matched HCs (8 males, 4 females; 57.8 ± 4.7 years) served as controls. No subjects had previously participated in an EEG experiment. The Institutional Review Boards of the Samsung Medical Center (Application Number: SMC 2013-02-091) and Korea Institute of Science and Technology (Application Number: KIST 2013–009) approved this study. The participants were informed about the study’s purpose, experimental procedures, and their right to withdraw at any time. Written informed consents were obtained from all of the participants. All of the research data were collected and analyzed under Institutional Review Board guidance.

**Table 1:** Clinical data of patients with chronic stroke

No	Age	Sex	AH	Diagnosis	Duration (months)	Grasp strength (kg)		Purdue Pegboard Test		FMA-UE	
						AH	UH	AH	UH	AH	UH
Supratentorial lesion including M1											
1	52	F	Rt.	Lt. MCA territory infarction	60	0	14.6	2	15	52	62
2	53	M	Lt.	Rt. MCA territory infarction	61	NT	23.33	NT	13	32	66
3	59	M	Lt.	Rt. MCA infarction	55	15.33	35.33	9	15	48	64
4	41	M	Rt.	Lt. MCA infarction	53	14	22	8	12	59	65
Mean	51.3	M	Lt.		57.3	9.8	23.8	6.3	13.8	47.8	64.3
(±SD)	(±7.5)	(75 %)	(50 %)		(±3.9)	(±8.5)	(±8.5)	(±4.4)	(±1.5)	(±11.4)	(±1.7)
Supratentorial lesion excluding M1											
5	56	F	Lt.	Rt. CR infarction	50	0	2.5	4	11	56	65
6	60	M	Lt.	Rt. thalamus, IC infarction	32	8	26.66	10	13	54	64
7	65	M	Rt.	Lt. BG ICH	117	5	21	13	12	53	63
8	46	F	Rt.	Lt. BG infarction	16	1	16	7	15	54	66
Mean	56.8	M	Lt.		53.8	3.5	16.5	8.5	12.8	54.3	64.5
(±SD)	(±8.1)	(50%)	(50%)		(±44.4)	(±3.9)	(±10.3)	(±3.9)	(±1.7)	(±1.3)	(±1.3)
Infratentorial lesion											
9	58	M	Rt.	Lt. medial medullary infarction	62	NT	23.3	6	14	31	66
10	49	M	Lt.	Rt. medial medullary infarction	48	6	36	2	15	44	65
11	57	M	Rt.	Lt. pontine infarction	67	8	27.6	6	12	40	66
12	52	M	Lt.	Rt. pontine infarction	37	11.33	23.66	5	10	45	66
Mean	54.0	M	Lt.		53.5	8.4	27.6	4.8	12.8	40.0	65.8
(±SD)	(±4.2)	(100 %)	(50 %)		(±13.6)	(±4.8)	(±5.9)	(±1.9)	(±2.2)	(±6.8)	(±0.5)
Mean	54.0	M	Lt.		54.8	6.9	22.7	6.5	13.1	47.3	64.8
(±SD)	(±6.6)	(75 %)	(50 %)		(±24.4)	(±5.7)	(±9.0)	(±3.7)	(±1.7)	(±9.2)	(±9.2)

Abbreviations: *FMA-UE* Fugl-Meyer Assessment Upper Extremity; *AH* Affected Hand, *UH* Unaffected Hand; *CR* Corona Radiata; *MCA* Middle Cerebral Artery; *IC* Internal Capsule; *BG* Basal Ganglia; *ICH* Intra Cerebral Hemorrhage

### Experimental protocol and EEG data processing

In this study, the subjects were asked to conduct grasping and supination movements with the affected hand; these are two basic hand functions involved in activities of daily living. They performed each movement with active, passive, and motor imagery (MI) tasks. In the active task, subjects were asked to perform a given movement with motor intention by themselves. A robotic device performed the movement in the passive task. In the MI task, each subject was asked to imagine the movement with motor intention, but he or she did not perform the physical movement. The experimental protocol consisted of three motor tasks, each composed of three blocks (nine blocks in total). Each block consisted of 14 repeated trials, and each trial consisted of four time periods: relax, motor task, stay, and return. A fixation appeared on the screen during the relax period with a random duration between 2 and 3 s. Participants performed a motor task in the 2-s motor task period, which started with auditory and visual cues. The 1-s stay period is necessary in order to prevent the risk of a sudden movement change. Then, the robotic device was reset to its original handle position during the return period in the case of active and passive motor tasks. Therefore, each participant performed 42 sequential trials (14 trials for each of the three blocks) for each of the three motor tasks (active, passive, and MI), accounting for a total of 126 trials; EEG data were recorded during the entire experimental protocol.

EEG signals were acquired with a 64-channel EEG active electrode system (sampling rate: 2,048 Hz; Active-two, BioSemi S.V., Amsterdam, Netherlands). The acquired EEG signals were preprocessed using the following steps: downsampling, 1–80 Hz band-pass and 60 Hz notch filtering, trial epoching, independent component analysis (ICA) for electrooculographic and muscle artifacts removal [30], and common average reference (CAR) [31]. In our study, the CAR was used for re-reference with the average of whole EEG channels for each individual EEG channel. Alternatively, the Laplacian montage method can be used when the local average surrounding a target EEG channel is adopted to adjust the bias of the target channel [32]. After preprocessing, spectral power was computed using short-time Fourier transform with a 500-ms hamming window, and sliding by 50 ms for each of the 64 EEG channels. The baseline of each epoch was defined as the 1 s before the motor task cues. The spectral power was normalized by subtracting the baseline mean from each data point in an epoch and by dividing the resulting value by the baseline SD. The ERD/ERS was defined as the spectral power changes in the motor task period relative to the baseline. Two frequency bands selected in our study include the mu (8–13 Hz) and beta (13–32 Hz) bands, both of which reflect sensorimotor rhythms. Detailed information on the experimental protocol and the EEG processing method can be found elsewhere [20].

The quantitative analyses of the EEG data were based on the LC and topographic mapping of the EEG spectral power. The hemispheric asymmetries for ERD/ERS, LC was calculated as follows:

1 $$ \mathrm{L}\mathrm{C}=\left(C-I\right)/\left(C+I\right) $$

where *C* denotes the ERD/ERS of the contralateral motor cortex and *I* denotes the ERD/ERS of the ipsilateral motor cortex [23, 33].

We compared the LC values across different combinations of the frequency bands (mu and beta bands), motor tasks (active, passive, and MI tasks), movements (supination and grasping movements), and participants (SM1+, SM1-, INF, all patients, and HCs). We observed the LC pattern in the mu and beta bands because these bands are known to be associated with motor movement.

In the analysis of this study, we focused on the active and MI motor tasks because the passive motor task using a robot-guided device would lack of the subject’s motor intention, a key factor in effective rehabilitation [34, 35]. ERD/ERS patterns on the active and MI motor tasks was compared between the subgroups of patients and the HCs. More specifically, Pearson’s linear correlation analysis was performed using the ERD/ERS power changes in the bilateral motor cortex during the motor task period. For the topographical analysis, we selected 28 EEG channels around the bilateral motor cortex and supplementary motor area (SMA), both of which are associated with motor movement. For each of the 28 channels, the ERD/ERS power changes were averaged across all HCs or each of the patient subgroups. In addition, we compared the EEG topographies from all possible combinations across the two frequency (i.e., mu and beta) bands, three motor tasks (i.e., active, passive and MI tasks), two different movements (i.e., grasping and supination), and four subject groups (i.e., SM1+, SM1-, INF and HCs). A Pearson’s correlation coefficient was calculated using the ERD/ERS power changes between the HCs and each of the three patients subgroups for each of the 28 channels. Then, one-way ANOVA was performed across the three patient subgroups using the 28 correlation coefficients across the 28 channels from each subgroup.

## Results

### Comparison of the LC patterns between all patients and HCs

**Fig. 1:**
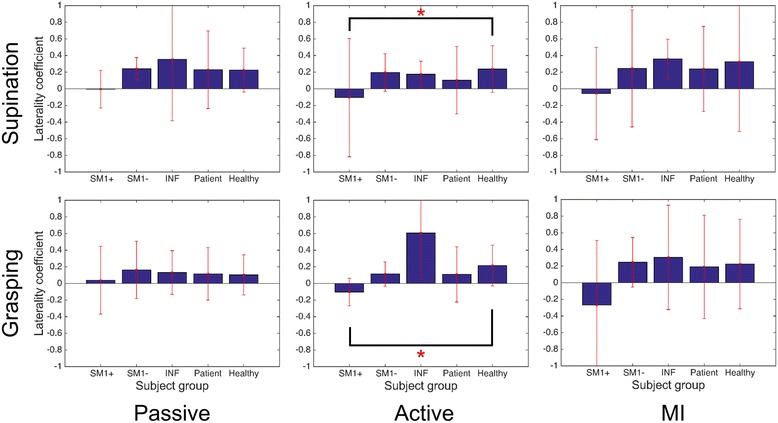
Beta band laterality coefficients for the three motor tasks (passive, active, and MI) in supination and grasping movements. Solid bars indicate the mean value; error bars reflect standard deviation. Significant results of pairwise statistical analysis on differences in laterality coefficients are indicated (rank sum test, *p < 0.05). Abbreviations: *SM1+* supratentorial lesion including M1; *SM1-* supratentorial lesion excluding M1; *INF* infratentorial lesion, Patient, all patients; Healthy, healthy controls

Figure 1 illustrates a comparison of the LC values in the beta band of patients and controls. The five bars indicate the LC values of SM1+, SM1-, INF, all patients, and HCs. The LC pattern in the mu band is displayed Additional file 1: Figure S1 in the additional material. In HCs, the ERD in the contralateral motor cortex was stronger than that in the ipsilateral motor cortex regardless of the movement and task types, which resulted in positive LC values.

The difference in the LC values between all patients and HCs was not significant, even though all the patients represented lower LC values compared to the controls in the active and MI tasks.

### Comparison of LC patterns between patient subgroups

Figure 1 shows that the SM1+ subgroup had a negative LC value in both of the movements in the active and MI motor tasks. Especially in the active task, there were significant differences between the SM1+ subgroup and HCs (rank-sum test, *p* < 0.05). The SM1- and INF subgroups had positive values in the same condition. For the passive task, LC values were very small values. It indicates the brain activation in bilateral motor cortex. The SM1+ subgroup exhibited negative LC values while they performed the MI task; however, these values were not significantly different from those of the other groups.

### Comparison of the EEG responses relative to the lesion locations in the patients

**Fig. 2:**
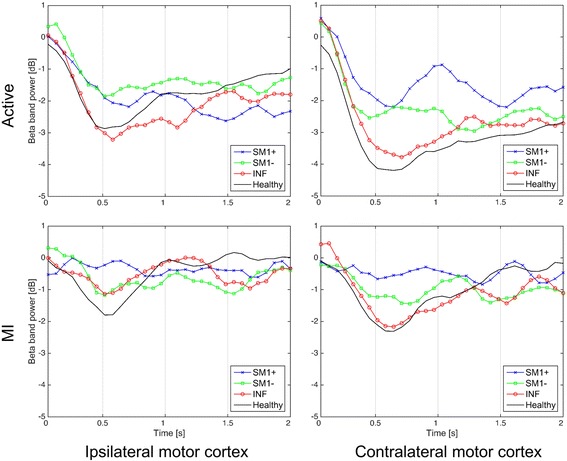
Average power patterns of the beta band in the ipsilateral and contralateral motor cortex during 2 s of active and MI supination movements. The ipsilateral motor cortex is in the unaffected hemisphere, and the contralateral motor cortex is in the affected hemisphere in patients

Figure 2 shows the average power patterns of the beta band of the three patient subgroups and HCs during the 2 s supination movements in the active and MI tasks. The average power patterns of the beta band showed marginal differences between the ipsilateral and contralateral sides of the motor cortex and between the active and MI tasks. The ERD in the contralateral motor cortex was generally stronger than that in the ipsilateral motor cortex. The ERD of the HCs appeared stronger than those of the patient subgroups, except in the ipsilateral motor cortex during the active task.

Table 2 lists Pearson’s linear correlation coefficients of the average beta band power (shown in Fig. 2) calculated between each patient subgroup and the HCs. In most cases, the correlation coefficients are statistically significant. Moreover, the correlation coefficients consistently decreased in the following order: INF > SM1- > SM1 + .

**Table 2:** Pearson’s linear correlation coefficients between the average beta band power patterns of each subgroup and that of the HCs during supination movement

	Ipsilateral motor cortex			Contralateral motor cortex		
	INF	SM1-	SM1+	INF	SM1-	SM1+
Active	0.880^**^	0.676^**^	0.332	0.977^**^	0.824^**^	0.802^**^
MI	0.511^**^	0.263	-0.470^**^	0.771^**^	0.388^*^	0.176

^*^ *p* < 0.05, ^**^ *p* < 0.01

Abbreviations: *INF* infratentorial lesion; *SM1-* supratentorial lesion excluding M1; *SM1+* supratentorial lesion including M1

### Topographical analysis

**Fig. 3:**
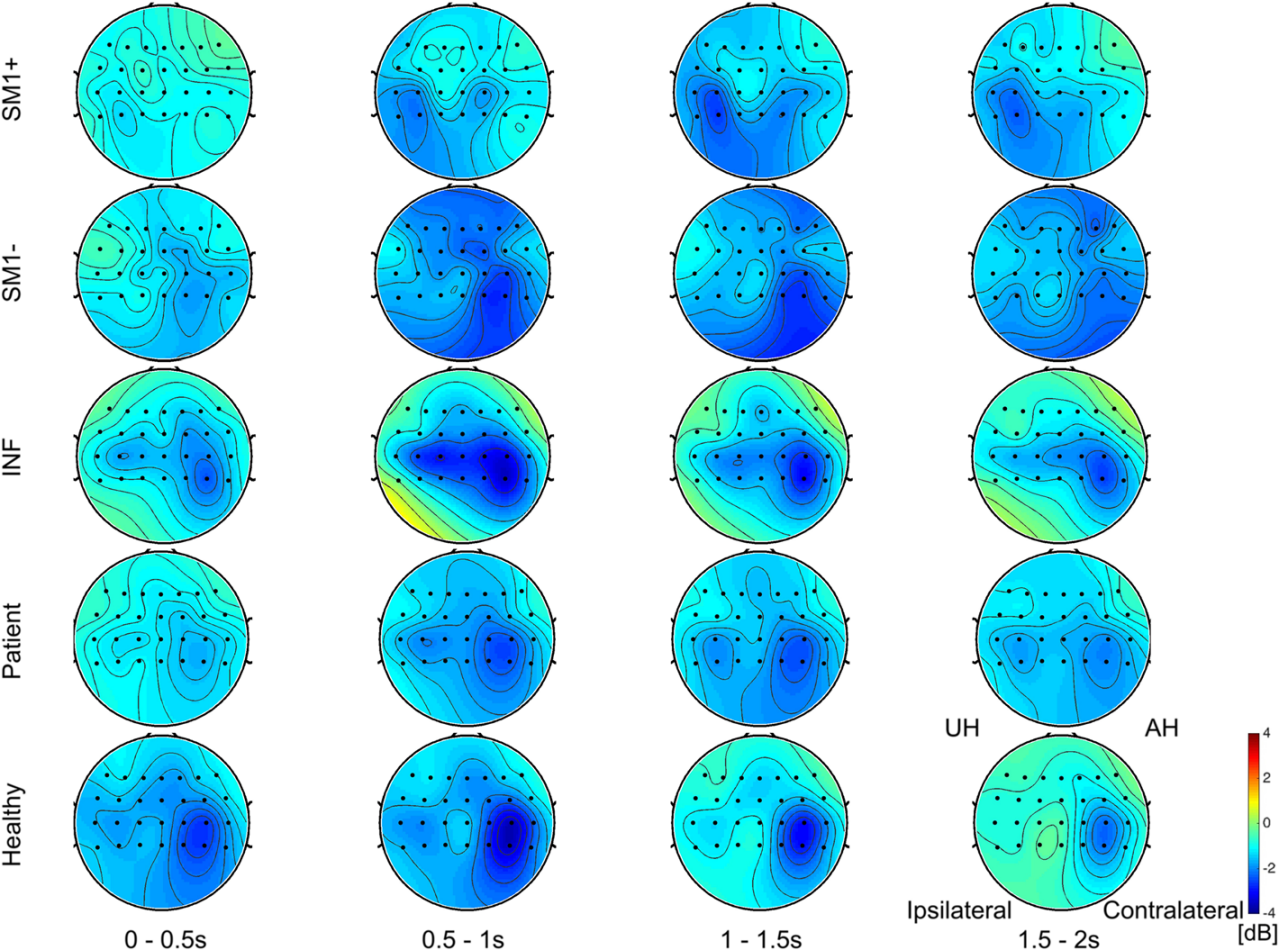
Twenty-eight channel topography of the beta band during active supination movement. The horizontal axis represents 2 s of the motor task with a 0.5-s window interval. The vertical axis represents the subject groups. The upper three rows represent each subgroup of patients according to their lesion location. The fourth row represents all patients and the last row represents the healthy controls

A topographical analysis was implemented based on the 28 EEG channels around the SMA and bilateral motor cortex. Figure 3 shows the average beta band power distributions across the subjects in each group during the supination movement in the active task. The topographies in the MI task are shown in Additional file 2: Figure S2 in the additional material. The upper three rows display the topography patterns that corresponded to the three patient subgroups. For the SM1+ subgroup (first row), the ERD of the ipsilateral side was stronger than that of the contralateral side. For the SM1- subgroup (second row), the ERD of the contralateral side was stronger than that of the ipsilateral side, and it was particularly widespread. The INF subgroup (third row) showed that the ERD of the contralateral side was stronger than that on the ipsilateral side, and, in particular, the ERD distribution was focused on the motor cortex and parietal area on the contralateral side. For all of the patient subgroups in the fourth row, the ERD distribution was located in the bilateral motor cortex. In the case of the HCs in the last row, the ERD of the contralateral side was stronger than that of the ipsilateral side, and the strong ERD distribution was focused on the contralateral motor cortex.

**Fig. 4:**
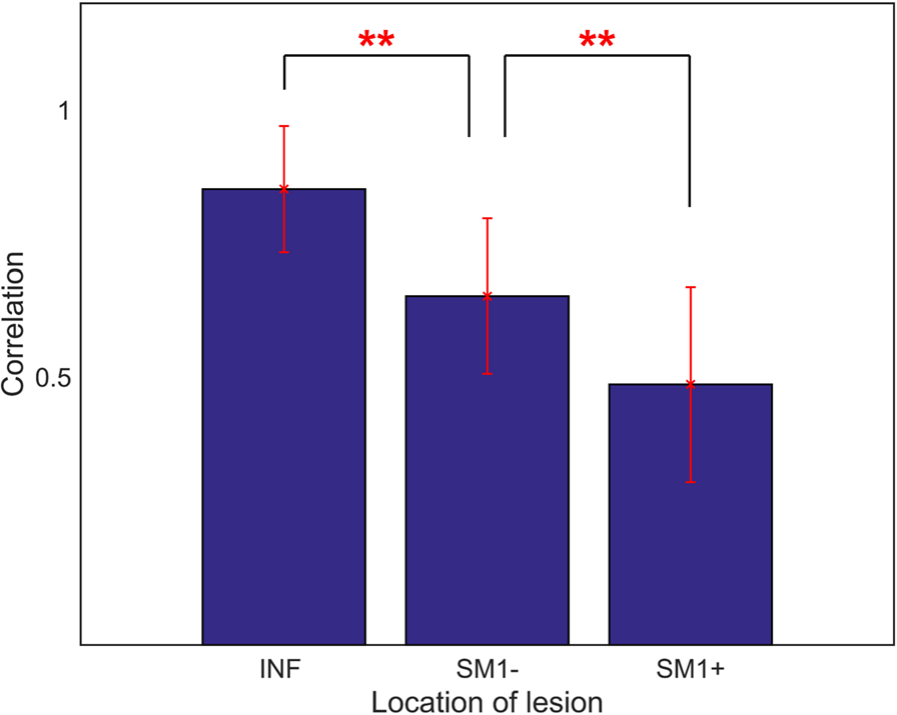
Pearson’s correlation coefficients for the beta band power changes between the HCs and each of the three patient subgroups for each of the 28 channels during the active task supination movement. Significant results of a pairwise statistical analysis on the differences in the correlation coefficients are indicated (one-way ANOVA test, **p < 0.01)

Figure 4 shows the similarities of the beta band power changes across the 28 channels between the HCs and each of the three patient subgroups. The INF group showed similar ERD/ERS power changes in comparison to HCs, whereas the SM1+ group was represented a deviated ERD/ERS power changes compared to the HCs. The correlation coefficients differed significantly between the three subgroups (one-way ANOVA test, **p < 0.01). In the case of MI task, the similarities of the beta band power changes across the 28 channels between the HCs and each of the three patient subgroups are shown in Additional file 3: Figure S3 in the additional material.

## Discussion

In this study, we investigated how EEG patterns differ across the stroke patient groups divided by lesion location, while they performed motor tasks, such as active, passive, and MI tasks with both supination and grasping movements. The active and MI tasks require the subject’s motor intention, whereas the passive task does not. The active and passive tasks are performed with the physical movement, but the MI task does not. Moreover, the LC values of the ERD in the left and right motor areas were statistically different between patient subgroups and the HCs in the beta band (Fig. 1); however, there were no significant differences in the mu band (Additional file 1: Figure S1 in the additional material).

The supination and grasping movements show very similar ERD/ERS patterns. The temporal patterns of the average beta band power and the topographic distribution of the beta band during these two movement types are reported in Additional file 4: Figure S4 and Additional file 5: Figure S5, respectively. Moreover, Fig. 1 shows that supination and grasping movements have similar LC values. This might be because of due to the similarity of sensorimotor EEG changes and topography between the two movements. Therefore, we examined the results for sensorimotor EEG changes and topography analysis only for the supination movements.

For the SM1+ group in the active and MI tasks, the LC value was always negative in both the grasping and supination movements (Fig. 1). This indicated that the ERD power in the ipsilateral motor cortex was stronger than that in the contralateral motor cortex. The contralateral motor cortex of the SM1+ patients was directly damaged, and therefore, was no longer capable of normal motor function. Instead, the unaffected ipsilateral motor cortex assumed the function of the damaged area [28].

The SM1- and INF groups showed positive LC values in the same tasks. In these groups, the motor cortex was not directly damaged; therefore, it showed a level of brain activation similar to that observed in HCs. Interestingly, in the passive task, the SM1+ group exhibited an LC value close to zero in the supination movement and a low positive LC value in the grasping movement. These results suggested that the participant’s motor intention, which was required in the active and MI tasks, might have resulted in a strong ERD in the ipsilateral motor cortex.

For the HCs, the LC values were positive in all of the tasks. These results were similar to those of the study by Kaiser [36]. She investigated sensorimotor EEG changes during passive, active, and MI tasks in healthy elderly individuals. Interestingly, in both movement tasks and bilateral motor cortex, there is a consistent trend in the correlation coefficients between each subgroup and HCs, whose values consistently decreased in the following order: INF > SM1- > SM1+ (Table 2). In addition, we measured how the beta band power changed during the active supination movement task in the 28 EEG channels around the motor cortex that were selected for the topographical analysis. Figure 4 shows the correlation coefficients between each patient subgroup and the HCs; the statistically significant differences observed among the three coefficients pairs are also shown (one-way ANOVA, *p* < 0.01). From these results, we can conclude that the similarity between the beta band power patterns is the highest between INF and HCs and the lowest between SM1+ and HCs.

In TMS studies, cortical lesion groups show properties that differ in similar ways from those of the subcortical and HCs. Shimizu et al. compared intracortical inhibition (ICI) and transcallosal inhibition (TCI) in cortical and subcortical lesion groups [37]. They demonstrated that ICI was significantly reduced in the cortical lesion group compared with the age-matched HCs. TCI was absent in the cortical lesion group, but it was observed in the subcortical lesion and HCs. Liepert et al. compared the properties of four groups (motor cortex, striatocapsular, internal capsule, and pontine lesions) and demonstrated that only the motor cortex lesion group had a loss of the ICI in the affected hemisphere [13].

As shown in Fig. 3, the topography analysis showed distinct differences between the three subgroups of patients. The INF group with lesions in the deepest location showed EEG topographical maps that were similar to those of HCs. The ERD was stronger around the contralateral motor cortex than around the ipsilateral motor cortex, and the ERD distribution was focused on the motor cortex and parietal area on the contralateral side. The SM1+ and SM1- groups showed topographies that differed distinctly from the INF group and HCs. The SM1+ group had a strong and focused ERD distribution on the ipsilateral side, and the SM1- group showed a widespread ERD distribution.

We inferred that the interhemispheric inhibition (IHI) was associated with the different patterns of the topographical distributions that depended on the depth of the lesion location. IHI involves inhibitory interactions between the bilateral primary motor cortexes [38, 39].

Because the IHI in the SM1+ group decreased from the ipsilesional M1 to the contralesional M1, the ERD on the ipsilesional side may be stronger than that on contralesional side. This hypothesis is supported by the results of the study by Bütefisch et al. [40]. They reported that IHI decreased abnormally from the ipsilesional M1 to the contralesional M1 in the cortical lesion group but not in the subcortical lesion group.

The SM1- and INF groups had subcortical lesions that injured the pyramidal tract [41]. Thus, the injury does not greatly affect the IHI between the bilateral M1s [40]. We inferred that this was why the SM1- and INF groups had different patterns of neural activation compared with the SM1+ group.

As far as we are aware, subcortical lesions have not been specifically segmented in most lesion studies [17, 36, 40]. However, our study divided the subcortical lesion group into two subgroups and demonstrated that the beta band ERD distribution of the INF group was stronger and more focused in the ipsilesional hemisphere than that in the SM1- group. Nevertheless, the motor function of the INF group was more severely affected compared with the SM1- group. Because the neural mechanisms associated with the SM1- and INF lesions are not yet fully understood, additional studies investigating this issue, including ones using a simultaneous EEG-fMRI modality, are warranted [42].

Our results indicated that plasticity changes that occurred during the motor rehabilitation period differed depending on lesion location and that these changes produced different patterns of neural activation in patients with chronic stroke with different lesion locations. Our findings may be limited by the number of patients in each subgroup, and thus, a future study is warranted to investigate these findings in a large cohort.

## Conclusions

Previous studies have reported that ERD in patients with stroke occurs bilaterally during the same task [23–25]. In our study, we observed similar results in all patient subgroups. However, in patient subgroups that were classified by their different lesion locations, we observed distinctly different beta band EEG patterns in each group. These findings indicated that EEG spectral analyses should be implemented for patients with stroke considering their lesion location. We envision that this finding will provide an important foundation for studies of BCI-based motor rehabilitation.

## Additional files

Additional file 1: Figure S1. Mu band laterality coefficients for the three motor tasks (passive, active, and MI) in supination and grasping movements. Solid bars indicate mean values and the error bars indicate standard deviation. Abbreviations: *SM1+* supratentorial lesion including M1; *SM1-* supratentorial lesion excluding M1; *INF* infratentorial lesion, Patient, all patients; Healthy, Healthy controls (JPG 835 kb) Additional file 2: Figure S2. Twenty-eight channel topography of the beta band in MI supination movement. The horizontal axis represents 2 s of the motor task with a 0.5-s window interval. The vertical axis represents the participant group. The upper three rows represent each subgroup of patients according to their lesion location. The fourth row represents all patients and the last row represents the healthy controls. (JPG 2440 kb) Additional file 3: Figure S3. Pearson’s correlation coefficients for the beta band power changes between the HCs and each of the three patient subgroups for each of the 28 channels during the MI task supination movement. Significant results of a pairwise statistical analysis on the differences in correlation coefficients are indicated (one-way ANOVA test, **p* < 0.05; ***p* < 0.01). (JPG 640 kb) Additional file 4: Figure S4. Average patterns of the beta band power in the contralateral motor cortex during 2 s of active task in supination (left side) and grasping (right side) movements. (JPG 780 kb) Additional file 5: Figure S5. Twenty-eight channel topography of the beta band during active supination (left side) and grasping (right side) movements. The horizontal axis represents 2 s of the motor task with a 0.5-s window interval. The vertical axis represents the subject groups. The upper three rows represent each subgroup of patients according to their lesion location. The fourth row represents all patients and the last row represents healthy controls. (JPG 1727 kb)

## Abbreviations

fMRI: Functional magnetic resonance imaging
EEG: Electroencephalographic
SM1+: Supratentorial lesions that included M1
SM1-: Supratentorial lesions that excluded M1
INF: Infratentorial
MI: Motor imagery
LC: Laterality coefficient
ERD: Event-related desynchronization
ERS: Event-related synchronization
TMS: Transcranial magnetic stimulation
BCI: Brain-computer interface
SD: Standard deviation
HCs: Healthy controls
SMA: Supplementary motor area
ICI: Intracortical inhibition
TCI: Transcallosal inhibition
IHI: Interhemispheric inhibition

## References

[1] Murray CJ, Lopez AD (1997). Mortality by cause for eight regions of the world: Global burden of disease study. Lancet.

[2] Donnan G, Fisher M, Macleod M, Davis SM (2008). Stroke. Lancet.

[3] Crafton KR, Mark AN, Cramer SC (2003). Improved understanding of cortical injury by incorporating measures of functional anatomy. Brain.

[4] Shelton FN, Reding MJ (2001). Effect of lesion location on upper limb motor recovery after stroke. Stroke.

[5] Tatemichi TK, Desmond DW, Stern Y, Paik M, Sano M, Bagiella E (1994). Cognitive impairment after stroke: frequency, patterns, and relationship to functional abilities. J Neurol Neurosurg Psychiatr.

[6] Robinson RG, Kubos KL, Starr LB, Rao K, Price TR (1984). Mood disorders in stroke patients: importance of location of lesion. Brain.

[7] Bhogal SK, Teasell R, Foley N, Speechley M (2004). Lesion location and poststroke depression systematic review of the methodological limitations in the literature. Stroke.

[8] Liepert J, Bauder H, Miltner WH, Taub E, Weiller C (2000). Treatment-induced cortical reorganization after stroke in humans. Stroke.

[9] Chen R, Cohen LG, Hallett M (2002). Nervous system reorganization following injury. Neuroscience.

[10] Cauraugh JH, Summers JJ (2005). Neural plasticity and bilateral movements: a rehabilitation approach for chronic stroke. Progr Neurobiol.

[11] Feydy A, Carlier R, Roby-Brami A (2002). Longitudinal study of motor recovery after stroke recruitment and focusing of brain activation. Stroke.

[12] Schiemanck SK, Kwakkel G, Post MW, Kappelle JL, Prevo AJ (2008). Impact of internal capsule lesions on outcome of motor hand function at one year post-stroke. J Rehabil Med.

[13] Dimyan MA, Cohen LG (2010). Contribution of transcranial magnetic stimulation to the understanding of functional recovery mechanisms after stroke. Neurorehabil Neural Repair.

[14] Liepert J, Restemeyer C, Kucinski T, Zittel S, Weiller C (2005). Motor strokes the lesion location determines motor excitability changes. Stroke.

[15] Alexander LD, Black SE, Patterson KK, Gao F, Danells CJ, McIlroy WE (2009). Association between gait asymmetry and brain lesion location in stroke patients. Stroke.

[16] Luft AR, Forrester L, Macko RF, McCombe-Waller S, Whitall J, Villagra F (2005). Brain activation of lower extremity movement in chronically impaired stroke survivors. Neuroimage.

[17] Luft AR, Waller S, Forrester L (2004). Lesion location alters brain activation in chronically impaired stroke survivors. Neuroimage.

[18] Buch E, Weber C, Cohen LG (2008). Think to move: a neuromagnetic brain-computer interface (BCI) system for chronic stroke. Stroke.

[19] Ang KK, Guan C, Chua K (2011). A large clinical study on the ability of stroke patients to use an EEG-based motor imagery brain-computer interface. Clin EEG Neurosci.

[20] Park W, Kwon GH, Kim DH, Kim YH, Kim SP, Kim L (2015). Assessment of cognitive engagement in stroke patients from single-trial EEG during motor rehabilitation. IEEE Trans Neural Syst Rehabil Eng.

[21] Pfurtscheller G, Lopes Da Silva FH (1999). Event-related EEG/MEG synchronization and desynchronization: basic principles. Clin Neurophysiol.

[22] Wolpaw JR, Birbaumer N, McFarland DJ, Pfurtscheller G, Vaughan TM (2002). Brain–computer interfaces for communication and control. Clin Neurophysiol.

[23] Kaiser V, Daly I, Pichiorri F, Mattia D, Müller-Putz GR, Neuper C (2012). Relationship between electrical brain responses to motor imagery and motor impairment in stroke. Stroke.

[24] Rossiter HE, Borrelli MR, Borchert RJ, Bradbury D, Ward NS (2014). Cortical mechanisms of mirror therapy after stroke. Neurorehabil Neural Repair.

[25] Dimyan MA, Cohen LG (2011). Neuroplasticity in the context of motor rehabilitation after stroke. Nat Rev Neurol.

[26] Gong W, Zhang T, Shan L (2013). Cortical lateralization in stroke patients measured by event-related potentials during motor imagery. Mol Med Rep.

[27] Mathiowetz V, Kashman N, Volland G, Weber K, Dowe M, Rogers S (1985). Grip and pinch strength: normative data for adults. Arch Phys Med Rehabil.

[28] Mathiowetz V, Weber K, Kashman N, Volland G (1985). Adult norms for the nine hole peg test of finger dexterity. OTJR: Occupation Participation Health.

[29] Fugl-Meyer AR, Jääskö L, Leyman I, Olsson S, Steglind S (1975). The post-stroke hemiplegic patient. 1. a method for evaluation of physical performance. Scand J Rehabil Med.

[30] Vorobyov S, Cichocki A (2005). Blind noise reduction for multisensory signals using ICA and subspace filtering, with application to EEG analysis. Biol Cybern.

[31] Binnie CD, Cooper R, Mauguiere F, Osselton JW, Prior PF, Tedman B (2003). EEG, Paediatric neurophysiology, special techniques and applications.

[32] Gordon R, Rzempoluck EJ (2004). Introduction to laplacian montages. Am J Electroneurodiagnostic Technol.

[33] Pivik RT, Broughton RJ, Coppola R, Davidson RJ, Fox N, Nuwer MR (1993). Guidelines for the recording and quantitative analysis of electroencephalographic activity in research contexts. Psychophysiology.

[34] Daly JJ, Fang Y, Perepezko EM, Siemionow V, Yue GH (2006). Prolonged cognitive planning time, elevated cognitive effort, and relationship to coordination and motor control following stroke. IEEE Trans Neural Syst Rehabil Eng.

[35] Petrella L, McColl MA, Krupa T, Johnston J (2005). Returning to productive activities: Perspectives of individuals with long-standing acquired brain injuries. Brain Inj.

[36] Kaiser V, Kreilinger A, Müller-Putz GR, Neuper C (2011). First steps toward a motor imagery based stroke BCI: new strategy to set up a classifier. Front Neurosci.

[37] Shimizu T, Hosaki A, Hino T, Sato M, Komori T, Hirai S (2002). Motor cortical disinhibition in the unaffected hemisphere after unilateral cortical stroke. Brain.

[38] Ferbert A, Priori A, Rothwell JC, Day BL, Colebatch JG, Marsden CD (1992). Interhemispheric inhibition of the human motor cortex. J Physiol.

[39] Daskalakis ZJ, Christensen BK, Fitzgerald PB, Roshan L, Chen R (2002). The mechanisms of interhemispheric inhibition in the human motor cortex. J Physiol.

[40] Bütefisch CM, Wessling M, Netz J, Seitz RJ, Hömberg V (2008). Relationship between interhemispheric inhibition and motor cortex excitability in subacute stroke patients. Neurorehabil Neural Repair.

[41] Radlinska B, Ghinani S, Leppert IR, Minuk J, Pike GB, Thiel A (2010). Diffusion tensor imaging, permanent pyramidal tract damage, and outcome in subcortical stroke. Neurology.

[42] Kim HC, Yoo SS, Lee JH (2015). Recursive approach of EEG-segment-based principal component analysis substantially reduces cryogenic pump artifacts in simultaneous EEG-fMRI data. Neuroimage.

