# Supplementary figures and images for: EEG response varies with lesion location in patients with chronic stroke
Source: J Neuroeng Rehabil. 2016 Mar 2;13:21. doi: 10.1186/s12984-016-0120-2 (PMC4776402; doi:10.1186/s12984-016-0120-2)

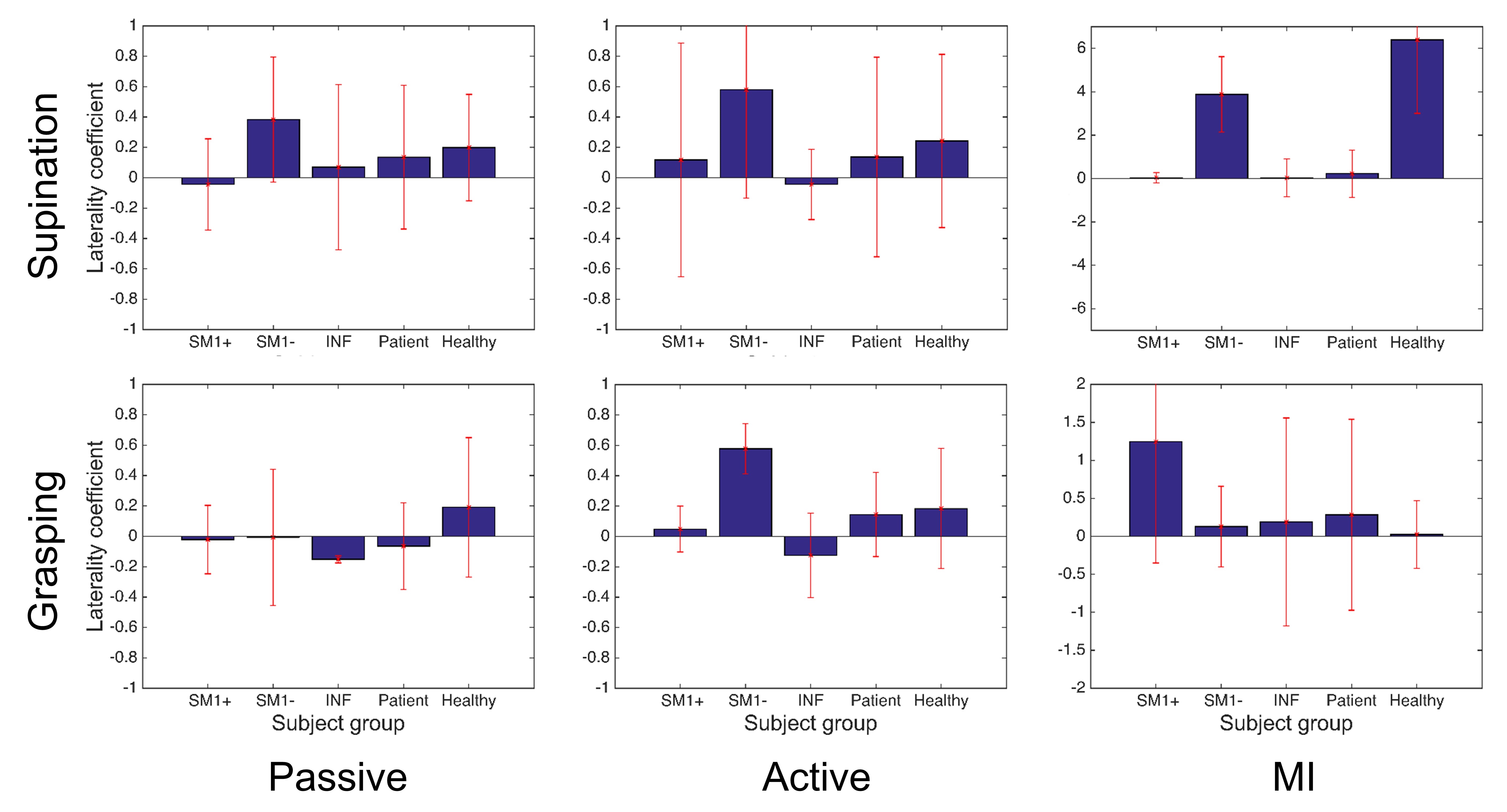

Supplement: Additional file 1: Figure S1. — Mu band laterality coefficients for the three motor tasks (passive, active, and MI) in supination and grasping movements. Solid bars indicate mean values and the error bars indicate standard deviation. Abbreviations: SM1+ supratentorial lesion including M1; SM1- supratentorial lesion excluding M1; INF infratentorial lesion, Patient, all patients; Healthy, Healthy controls (JPG 835 kb) [file 12984_2016_120_MOESM1_ESM.jpg]

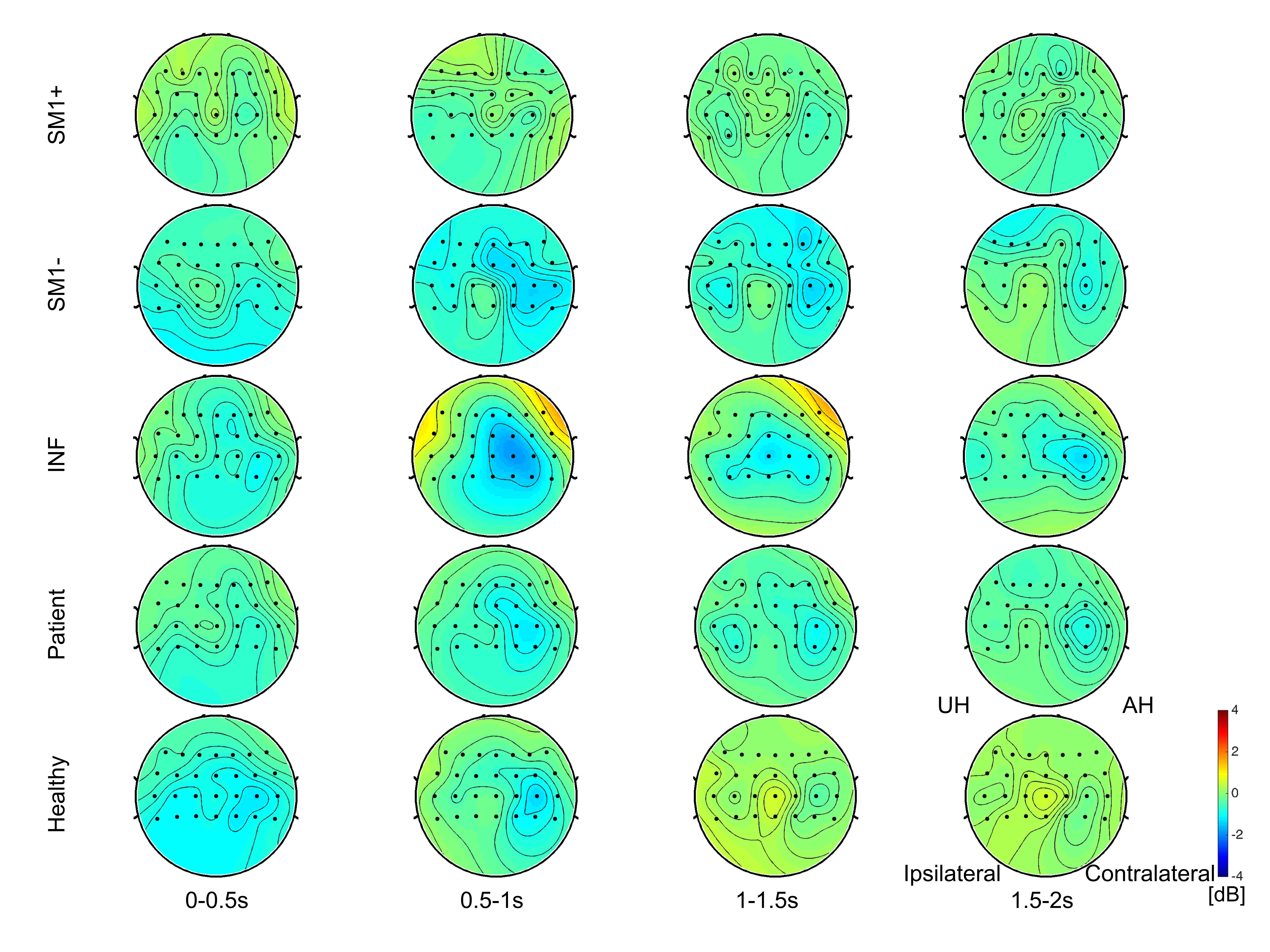

Supplement: Additional file 2: Figure S2. — Twenty-eight channel topography of the beta band in MI supination movement. The horizontal axis represents 2 s of the motor task with a 0.5-s window interval. The vertical axis represents the participant group. The upper three rows represent each subgroup of patients according to their lesion location. The fourth row represents all patients and the last row represents the healthy controls. (JPG 2440 kb) [file 12984_2016_120_MOESM2_ESM.jpg]

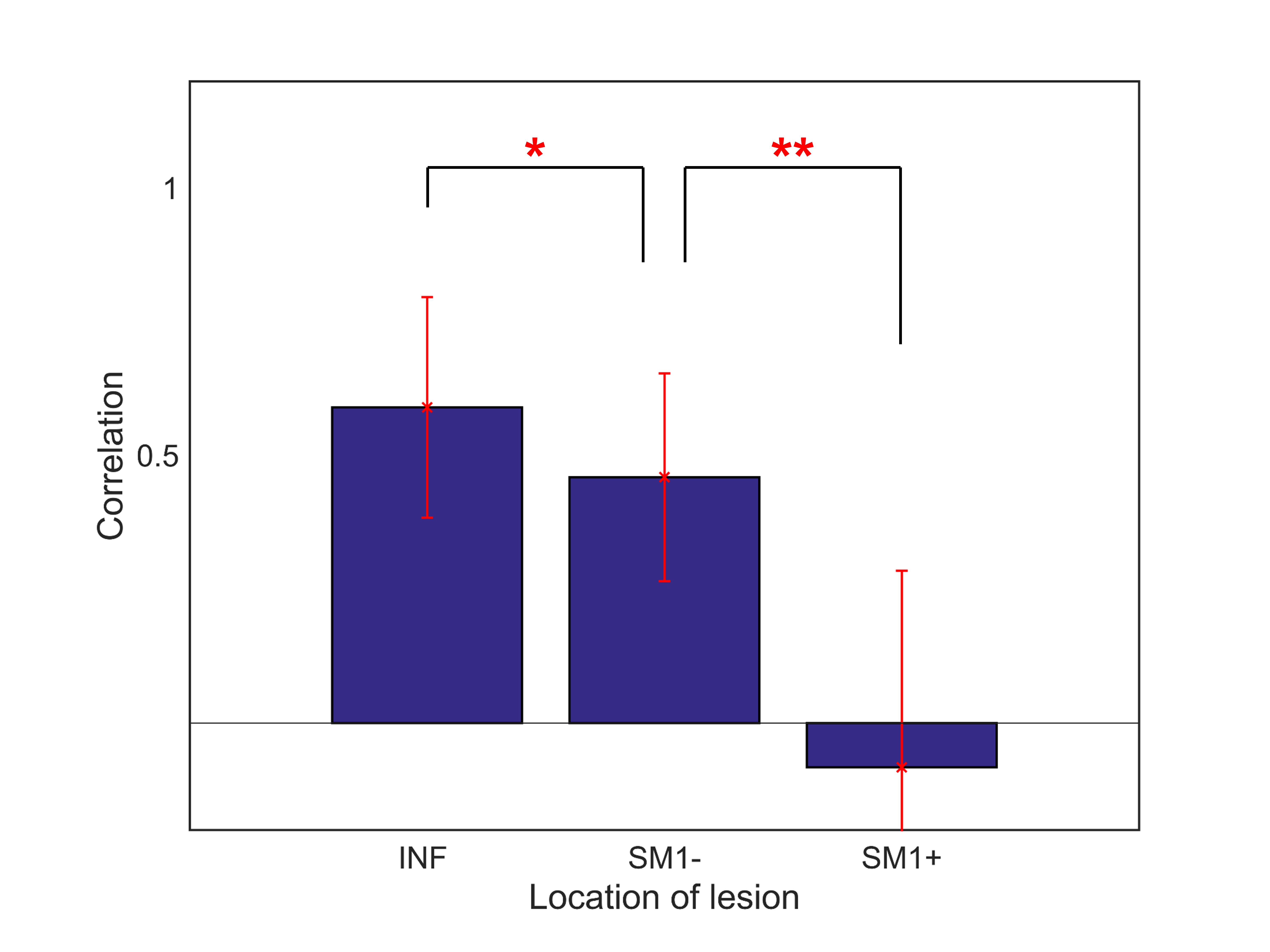

Supplement: Additional file 3: Figure S3. — Pearson’s correlation coefficients for the beta band power changes between the HCs and each of the three patient subgroups for each of the 28 channels during the MI task supination movement. Significant results of a pairwise statistical analysis on the differences in correlation coefficients are indicated (one-way ANOVA test, *p < 0.05; **p < 0.01). (JPG 640 kb) [file 12984_2016_120_MOESM3_ESM.jpg]

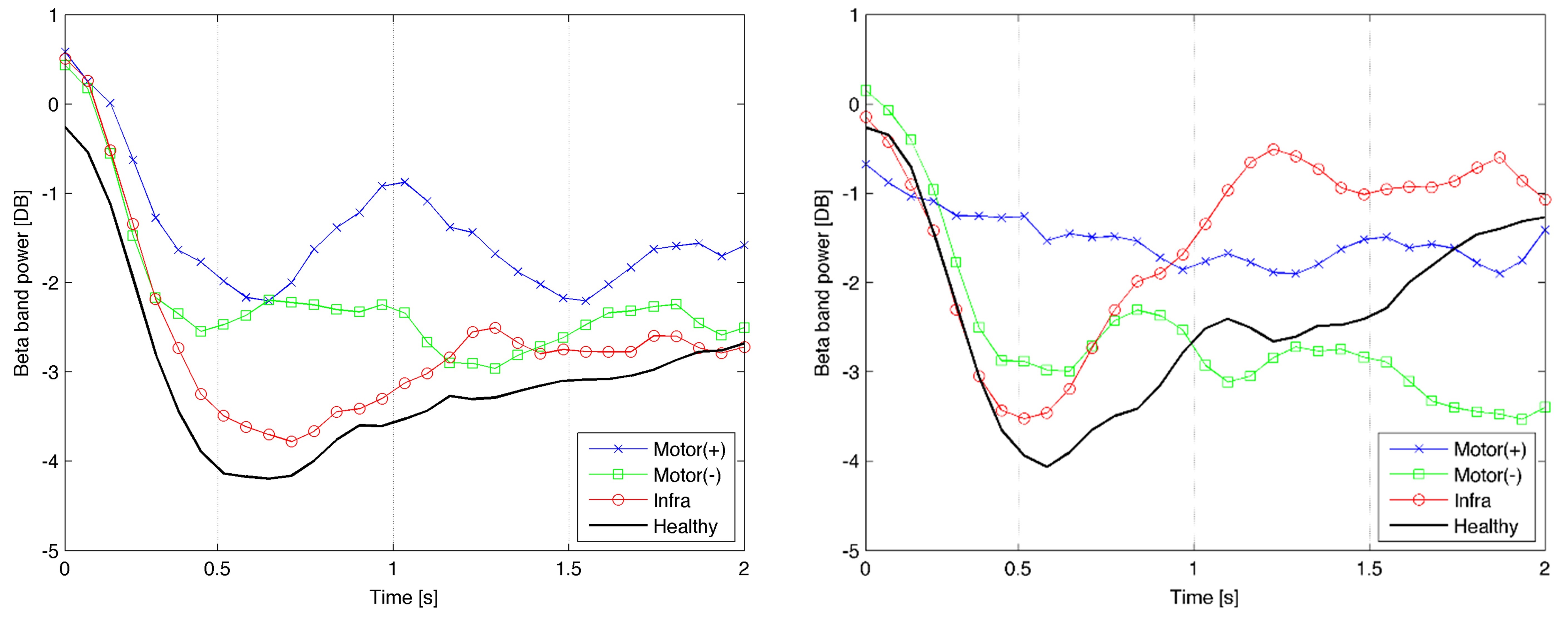

Supplement: Additional file 4: Figure S4. — Average patterns of the beta band power in the contralateral motor cortex during 2 s of active task in supination (left side) and grasping (right side) movements. (JPG 780 kb) [file 12984_2016_120_MOESM4_ESM.jpg]

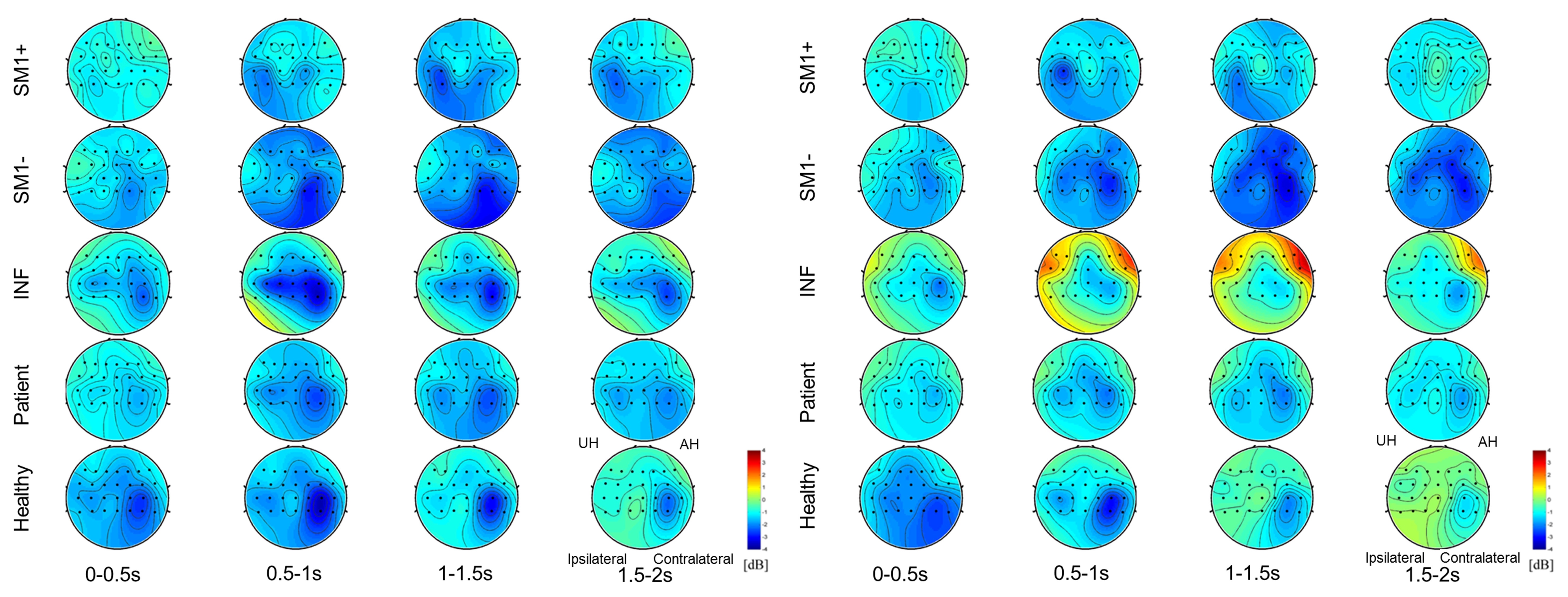

Supplement: Additional file 5: Figure S5. — Twenty-eight channel topography of the beta band during active supination (left side) and grasping (right side) movements. The horizontal axis represents 2 s of the motor task with a 0.5-s window interval. The vertical axis represents the subject groups. The upper three rows represent each subgroup of patients according to their lesion location. The fourth row represents all patients and the last row represents healthy controls. (JPG 1727 kb) [file 12984_2016_120_MOESM5_ESM.jpg]
